# Acute Vitreous and Intraretinal Hemorrhage with Multifocal Subretinal Fluid in Juvenile X-Linked Retinoschisis

**DOI:** 10.1155/2020/6638553

**Published:** 2020-11-24

**Authors:** Sidra Ibad, Carl S. Wilkins, Alexander Pinhas, Vincent Sun, Matthew S. Wieder, Avnish Deobhakta

**Affiliations:** ^1^Icahn School of Medicine at Mount Sinai, One Gustave L. Levy Place, New York, NY, USA; ^2^New York Eye and Ear Infirmary of Mount Sinai, 310 East 14th Street, Retina Center, New York, NY, USA

## Abstract

**Purpose:**

To report a rare case of spontaneous vitreous and intraretinal hemorrhage in a patient with juvenile X-linked retinoschisis which was managed conservatively.

**Methods:**

Single patient case report.

**Introduction:**

Juvenile X-linked retinoschisis (JXLR) most often occurs as a result of a genetic defect in the retinoschisin (RS1) gene, causing a separation between the ganglion cell layer and the nerve fiber layer. Spontaneous vitreous hemorrhage has been reported as an uncommon secondary consequence of JXLR. We present a case of spontaneous vitreous and diffuse macular intraretinal hemorrhages in a patient with JXLR which resolved with medical management alone.

**Results:**

A 23-year-old man with a history of juvenile X-linked retinoschisis presented to the ophthalmic emergency room complaining of acute onset of floaters in his right eye. On examination, the patient was found to have a new vitreous hemorrhage with diffuse intraretinal hemorrhages in his right eye, without new retinal tears or detachment. SD-OCT demonstrated multifocal pockets of subretinal fluid. The genetic testing panel revealed a hemizygous mutation in the RS-1 gene. He was managed conservatively on oral acetazolamide, with the resolution of the subretinal fluid and with both visual and symptomatic improvement.

**Conclusions:**

Spontaneous vitreous hemorrhage may rarely occur in patients with JXLR, even in the absence of acute retinal tear or detachment. This case demonstrates an atypical presentation of vitreous hemorrhage with diffuse intraretinal hemorrhage and new multifocal areas of subretinal fluid which improved without surgical intervention. Good outcomes may be achieved in these patients with conservative management alone, even in atypical presentations.

## 1. Introduction

With an estimated prevalence ranging from 1 : 15,000 to 30,000, juvenile X-linked retinoschisis (JXLR) is the most common pediatric-onset retinal degeneration. The condition is defined by a characteristic radiating pattern of foveal schisis in nearly all patients, often with a bimodal age distribution in infancy or school-age patients. Other findings include peripheral retinoschisis and pigmentary changes [1]. Complications may arise from vitreoretinal traction, particularly in areas of schisis cavities, including retinal tears and rhegmatogenous retinal detachments (RRD), spontaneous vitreous hemorrhage, and retinal fibrosis. These deleterious secondary findings most commonly present within the first decade of life [2]. There are several prior reports of vitreous hemorrhage in JXLR, either spontaneous or secondary to retinal tear or detachment, treated successfully with surgical intervention [2–4]. We report an atypical case of spontaneous vitreous hemorrhage in JXLR that presented with diffuse intraretinal hemorrhages and formation of multifocal pockets of subretinal fluid, which improved with conservative medical management alone.

## 2. Case Report

**Figure 1:**
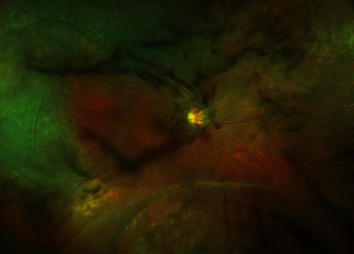
Widefield fundus photo of the right eye demonstrating intravitreal hemorrhage, vitreous debris, scattered intraretinal hemorrhage in the macula, and multiple areas of retinoschisis.

A 23-year-old man with a 6-year history of suspected JXLR treated with oral acetazolamide presented to the New York Eye and Ear Infirmary of Mount Sinai with a 3-day history of acute, painless decrease in vision with new floaters in his right eye (OD). Visual acuity on examination was stable from the prior exam at 20/60 bilaterally, with no relative afferent pupillary defect, and normotensive intraocular pressures bilaterally. The anterior segment examination was unremarkable. Dilated fundus examination revealed new vitreous debris with hemorrhage, stable areas of retinoschisis temporally and inferiorly with outer retinal holes and areas of vitreoretinal traction, and scattered intraretinal hemorrhages along the arcade within the macula and in the near periphery (Figure 1).

**Figure 2:**
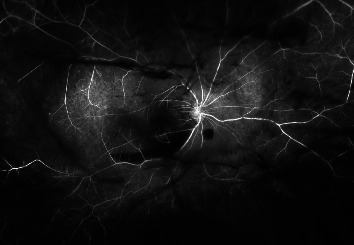
Widefield fluorescein angiography of the right eye demonstrating blocked fluorescence from vitreous debris and hemorrhage, with mild decreased perfusion at the areas of retinoschisis, and multiple areas of blocked fluorescence from intraretinal hemorrhage in the macula and near the periphery. There is no visible leakage.

An extrafoveal posterior vitreous detachment (PVD) was noted OD. Widefield fluorescein angiography demonstrated pinpoint areas of late leakage corresponding to new pockets of subretinal fluid, stable mild leakage from vessels at junctional areas of vitreoretinal traction, and nonperfusion at peripheral areas of schisis (Figure 2).

**Figure 3:**
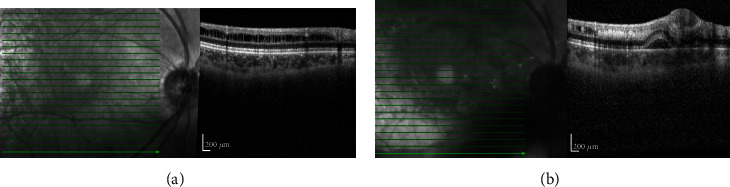
SD-OCT of the right eye at baseline (a; top) and after acute vitreous hemorrhage (b; bottom). (a) Retinoschisis in the inferior macula. (b) Intraretinal hemorrhage collecting within prior areas of schisis, with a focal serous neurosensory detachment.

Spectral domain ophthalmic coherence tomography (SD-OCT) demonstrated new, multifocal areas of subretinal cavitation with adjacent hyperreflective foci in the outer plexiform layer corresponding to areas of intraretinal hemorrhage on examination OD (Figures 3(a) and 3(b)).

No retinal tears or detachments were identified in either eye. The decision was made to closely observe the patient with medical management alone.

**Figure 4:**
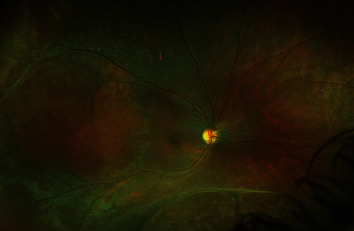
Widefield fundus photo of the right eye showing the resolution of vitreous hemorrhage, vitreous debris, and intraretinal macular hemorrhages.

Serial follow-up examinations revealed the clearing of the vitreous hemorrhage with the resolution of intraretinal hemorrhages (Figure 4).

**Figure 5:**
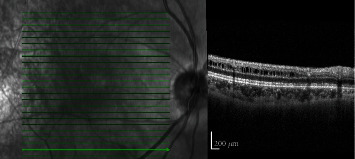
SD-OCT of the right eye demonstrating the resolution of subretinal fluid seen in Figure 3.

The peripheral retina remained stable without tears or detachment. Repeat SD-OCT showed the resolution of the subretinal fluid cavities and intraretinal hemorrhages (Figure 5).

The patient was continued on oral acetazolamide 125 mg twice daily, and visual acuity improved to 20/50. An inherited retinal degeneration gene panel (Spark Therapeutics Inc., Philadelphia, PA, USA) was obtained, which identified a hemizygous mutation in the retinoschisin (RS1) gene, confirming the diagnosis of JXLR. The patient remained stable at the 6-month follow-up on medical therapy.

## 3. Discussion

Vitreous hemorrhage is a known complication of JXLR, with or without retinal tear or detachment, and affects a minority of these patients. A wide range of management may be instituted, depending on whether a retinal break is detected or highly likely, and if a sufficient view exists to rule out those urgent etiologies. Though observational data is relatively sparse, vitreous hemorrhage or retinal detachment has been reported in up to 5% of patients with JXLR, almost always in the first decade of life [1]. These patients often are found to have abnormal vitreous, with 51% of patients reported to have vitreous veils and vitreoretinal traction [2]. Previous reports of vitrectomy in patients with JXLR demonstrated anatomic success in about 80% of surgeries, with coincident vitreous hemorrhage fairly rare at presentation, occurring in only 12% of these patients [3, 4]. We present a case of vitreous hemorrhage accompanied by atypical diffuse intraretinal hemorrhages and multifocal subretinal fluid, all of which were managed with medical management alone with a good outcome.

When vitrectomy is indicated for detachment in JXLR, chronic subretinal fluid or redetachment is common postoperatively, suggesting that despite the initial improvements, final anatomic success may be variable. While most surgeries lead to improved visual acuity, a considerable proportion experience no change or worsening of visual acuity [3, 4]. Vitrectomy remains the gold standard and may require retinectomy and internal limiting membrane (ILM) peeling to achieve reattachment. ILM peeling is controversial due to the formation of retinal breaks via fragile inner laminations but is recommended when surgically feasible. Inner retinal layer retinectomy may provide improved visualization of outer retinal layer breaks, relief of traction, and more complete vitreous removal; however, there is no proven difference in outcomes [5]. Vitreous hemorrhages without retinal breaks may spontaneously resolve; however, acute vitreous hemorrhages with retinal tear or detachment require urgent intervention. Nonsurgical treatments include laser photocoagulation at suspected areas of pathology and carbonic anhydrase inhibitors; however, level one data for these treatments is lacking. Though our patient suffered an acute vitreous hemorrhage at age 28, acute retinal pathology usually manifests in the first decade of life, with rare cases of vitreous hemorrhage reported in infancy [6, 7].

It is important to consider the implications of the genetics of JXLR and the atypical presentation of our patient, particularly in the context of the existing literature. X-linked retinoschisis has a high penetrance with variable phenotypic expression, which complicates the generalizability of interventional studies in guiding treatment paradigms, and may account for the variable outcomes between patients who appear similar anatomically [8]. Our patient developed hemorrhage into preexisting schisis cavities, as well as multifocal subretinal fluid cavities along the presumed areas of prior vitreoretinal traction which likely formed at the moment of release. There is sparse data regarding the rapidity of progression of detachment in these patients secondary to retinal breaks; hence, each patient may be managed differently based on presentation. Significant improvement in subretinal fluid without the presence of a retinal tear may be achieved with carbonic anhydrase inhibitors only, though the current data is limited to observational studies or case reports [9, 10].

Few case series of surgical interventions in these patients exist, though the most commonly reported reason for vitrectomy is RRD [3, 4]. Sen et al. reported the largest case series of 34 eyes with JXLR which underwent surgery for RRD, with only 11% presenting with concomitant vitreous hemorrhage, suggesting that most patients with detachment and JXLR do not present with this exam finding. Of note, nearly a third of patients in this series underwent a second surgery for redetachment, and 80% achieved final anatomic success, demonstrating overall worse outcomes in these patients compared to vitrectomy for more common causes of RRD [2]. Other interventional series present similar rates of vitreous hemorrhage (12-33%) in these patients, as well as the mean required number of surgeries (1.2-1.8), suggesting similar experience across institutions. The most common reason for anatomic failure in JXLR patients is the development of proliferative vitreoretinopathy [3, 4].

We present a case of JXLR in a 28-year-old male which was complicated by acute hemorrhagic posterior vitreous detachment with intraretinal hemorrhages within schisis cavities and multifocal, pinpoint serous retinal detachments managed with observation. We hypothesize that the prior vitreoretinal traction and abnormally friable tissue in this patient lead to the rupture of the deep capillary plexus during the completion of the PVD. Prior reports of surgical intervention for retinal detachment and vitreous hemorrhage indicate fair anatomic outcomes, though often with multiple procedures needed, and rarely with concomitant presence of vitreous hemorrhage. Without clear identification of a retinal tear in our patient, conservative management was chosen, and a good outcome was achieved without the need for surgery.

## Data Availability

No data were used to support this study.

## Abbreviations

JXLR: Juvenile X-linked retinoschisis
RRD: Rhegmatogenous retinal detachment
OD: Right eye
OS: Left eye
PVD: Posterior vitreous detachment
ILM: Internal limiting membrane
RS1: Retinoschisin 1
SD-OCT: Spectral domain ophthalmic coherence tomography.
